# Structural transformation induced by locked nucleic acid or 2′–O-methyl nucleic acid site-specific modifications on thrombin binding aptamer

**DOI:** 10.1186/1752-153X-8-19

**Published:** 2014-03-19

**Authors:** Bo Liu, Da Li

**Affiliations:** 1 Department of Laboratory Medicine, The First Hospital of China Medical University, Shenyang 110001, China

**Keywords:** G-quadruplex, TBA, LNA, 2*'*-OMeNA, Conformation

## Abstract

**Background:**

Locked nucleic acid (LNA) and 2*'*–O-methyl nucleic acid (OMeNA) are two of the most extensively studied nucleotide derivatives in the last decades. However, how they affect DNA quadruplex structures remains largely unknown. To explore their possible biological affinities for quadruplexes, we investigated how LNA- or OMeNA-substitutions affect G-quadruplex structure formation using a thrombin binding aptamer (TBA), the most studied extracorporal G-quadruplex-forming DNA sequence, which is frequently modified to increase its analytical performance.

**Results:**

The experimental results showed that when two or more nucleotides were substituted with LNA or OMeNA, the anti-parallel TBA structure was transformed into an unstructured random conformation in a 50 mM K^+^ environment; OMeNA appeared to have greater power to induce this transformation. However, the native TBA was unstructured in a 50 mM Ca^2+^ environment, whereas four or more LNA- or OMeNA- substitutions could convert this unstructured TBA into a parallel quadruplex structure. PAGE mobility measurements suggested that these TBAs might be a dimeric form.

**Conclusion:**

LNA or 2*'*-OMeNA site-specific modifications induced G-quadruplex structural transformation of TBA, which enriched our understanding of the intrinsic G-quadrupex forming property and affinity of LNA and OMeNA modifications. This study demonstrates possible applications in the regulation of gene expression (i.e. manual intervention of gene therapy), genetic analyses, molecular diagnosis and the construction of nano-scale biostructures.

## Background

**Figure 1:**
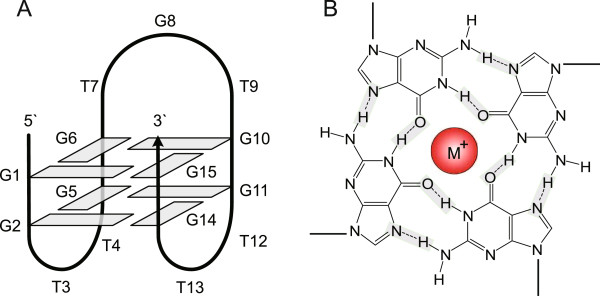
**Structure of thrombin binding aptamer G-quadruplex (A) and a G-tetrad plane (B).** B, M^+^ stands for positive metal ion.

A great number of nucleotide derivatives have been developed [1], which are different from natural DNA and RNA due to chemical modifications on either the phosphate group or the sugar moiety. Among them, locked nucleic acid (LNA) and 2′–O-methyl nucleic acid (OMeNA) have attracted great attention and have been extensively studied [2,3], since they demonstrate many advanced properties, such as the ability to increase duplex thermal stability and to recognize mismatched base pairs [4]. A previous study showed that these nucleotide derivatives can form Watson-Crick base pairs in complementary double strands [4]; beyond Watson-Crick hydrogen bonds, guanine-rich DNA and RNA sequences may fold into G-quadruplex structures through Hoogsteen hydrogen bonds [5-7], which may be involved in human biology and diseases [8]. These quadruplexes exist not only under natural conditions *in vivo*, but also have been investigated as therapeutic targets and applied as popular aptamers *in vitro*[5,9]. However, whether LNA- or OMeNA-modified sequences can also form Hoogsteen hydrogen bonds or quadruplex structures is not clear. To explore their possible biological affinities for quadruplexes, we investigated how LNA- or OMeNA-substitutions affect a well-defined G-quadruplex structure formed by the thrombin binding aptamer (TBA), the most commonly explored extracorporal aptamer [10]. TBA is an oligonucleotide of d(GGTTGGTGTGGTTGG) [11], which retains an anti-parallel, chair-like intra-molecular quadruplex structure in a K^+^ environment (Figure 1A) [12,13]. Two stacked G-tetrads, each of which is composed of four guanine residues through Hoogsteen hydrogen bonds (Figure 1B), are linked through three lateral loops. In this study, we used circular dichroism (CD) spectra and non-denatured PAGE to record and analyze the structural transitions and molecularities of native TBA and designed LNA- or OMeNA-modified TBAs (Table 1), both in K^+^ and Ca^2+^ ion environments, to investigate how LNA and OMeNA work in the formation of quadruplex structures.

**Table 1:** Primer oligonucleotide sequences of selected aptamers

**Molecule**^**[a]**^	**Nucleotide sequence**
TBA	5*'*-GGTTGGTGTGGTTGG-3*'*
g1 (2)	5*'*-G**g**TTGGTGTGGTTGG-3*'*
g1 (11)	5*'*-GGTTGGTGTG**g**TTGG-3*'*
g2 (2, 11)	5*'*-G**g**TTGGTGTG**g**TTGG-3*'*
g2 (5, 11)	5*'*-GGTT**g**GTGTG**g**TTGG-3*'*
g2 (10, 11)	5*'*-GGTTGGTGT**gg**TTGG-3*'*
g2 (11, 14)	5*'*-GGTTGGTGTG**g**TT**g**G-3*'*
g4 (2, 5, 11, 14)	5*'*-G**g**TT**g**GTGTG**g**TT**g**G-3*'*
g8 (1, 2, 5, 6, 10, 11, 14, 15)	5*'*-**gg**TT**gg**TGT**gg**TT**gg**-3*'*

^[a]^DNA and nucleotide derivatives are in upper and lower cases, respectively. N of gN represents the number of substitutions, and numbers in parentheses represent the modified positions marked in Figure 1A. In this case, g can be either L or M when the specified residues are substituted with LNA or OMeNA. In this study, a series of modified TBAs containing single, double, four or eight LNA or OMeNA substitutions at the designated positions (Table 1) were structurally analyzed. TBA g1 (2) and g1 (11) represent a single impactive substitution at position 2 or 11 of TBA, respectively. By the same token, TBA g2 indicates double substitutions with a specified combination: two diagonal positions 2 and 11 in the same tetrad plane g2 (2, 11), two adjacent positions in the same tetrad plane g2 (5, 11) and g2 (11, 14), and two stacked positions in two tetrad planes g2 (10, 11). TBA g4 and g8 symbolize four and eight substitutions in one and two tetrad planes, respectively.

## Results

### LNA- or OMeNA-modifications induced TBA structural transition from an antiparallel G-quadruplex to a random strand in a K^+^ environment

**Figure 2:**
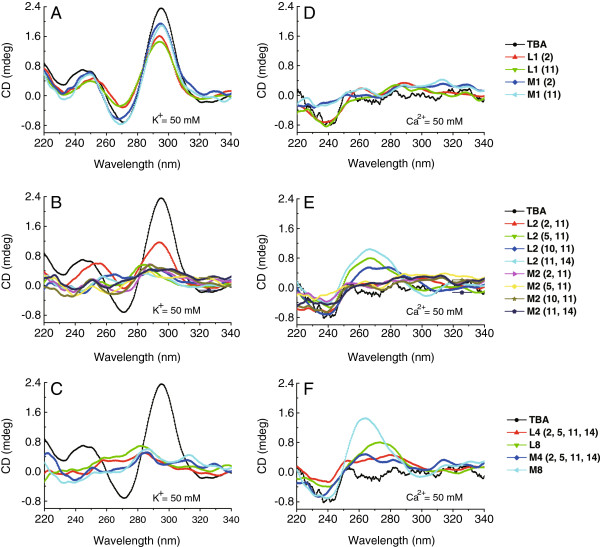
**CD spectra of natural TBA and LNA- or OMeNA-substituted TBAs. A**, single-substitution in K^+^; **B**, double-substitution in K^+^; **C**, four- and eight-substitution in K^+^; **D**, single-substitution in Ca^2+^; **E**, double-substitution in Ca^2+^; **F**, four- and eight-substitution in Ca^2+^. L or M stands for either LNA or OMeNA when the specified residues are substituted, N (N = 1 or 2 or 4 or 8) represents the number of substitutions, and numbers in parentheses represent the modified positions marked in Figure 1A.

To record quadruplex structure changes between native TBA and LNA- or OMeNA-modified TBAs, we used a circular dichroism spectropolarimeter to measure the sample structures in solution, as the anti-parallel G-quadruplex structure of native TBA in K^+^ solution can be identified conveniently by its CD spectrum characterized by a positive maximum at 295 nm and a negative minimum at 265 nm [13-18]. As shown in Figure 2A, B and C, the same type of CD spectrum was displayed by native TBA in the presence of 50 mM K^+^, compared to LNA- or OMeNA-modified TBAs which exhibited diverse structural conformations. Under the same conditions, the single LNA-substituted L1 (2) and L1 (11) also showed a characteristic wave crest at ~295 nm and a wave trough at ~265 nm, an indication of an anti-parallel G-quadruplex structure [6]; only the CD amplitudes were smaller, which may reflect a decreased tetraplex population (Figure 2A). Also, L2 (2, 11) induced the same type of CD spectrum (Figure 2B), although the CD amplitudes were much smaller, but still maintained an anti-parallel conformation. The other double LNA- or OMeNA-substituted TBAs, and even four and eight LNA- or OMeNA-substituted TBAs, however, did not stabilize the tetraplex structure. In 50 mM K^+^ buffer, the double LNA-substitution at the diagonal positions of L2 (2, 11) showed a CD spectrum with a small difference from the natural TBA, suggesting a minor alteration of the native TBA structure (Figure 2B). In the arrangement with the substitutions adjacent to each other, CD spectra of L2 (5, 11), L2 (10, 11) and L2 (11, 14) characterized an unstructured TBA (Figure 2B). When one or two tetrads were fully substituted by LNA, the CD spectra of four and eight LNA-substituted TBAs, L4 and L8, indicated a complete collapse of the G-case (Figure 2C). On the other hand, OMeNA substitutions also induced remarkable structural changes in natural TBA. M1 (2) and M1 (11) still maintained the anti-parallel G-quadruplex structure, similar to L1 (2) and L1 (11) (Figure 2A). However, two or more OMeNA substitutions completely transformed the native TBA into an unstructured topology (Figure 2B and C). These data demonstrate that both LNA and OMeNA substitutions can alter the TBA G-quadruplex structure, and OMeNA substitutions might have more destructive power than LNA to convert TBA into a structureless form in a K^+^ environment.

### LNA- or OMeNA-modifications tend to form a parallel G-quadruplex topology of TBA in a Ca^2+^ environment

**Figure 3:**
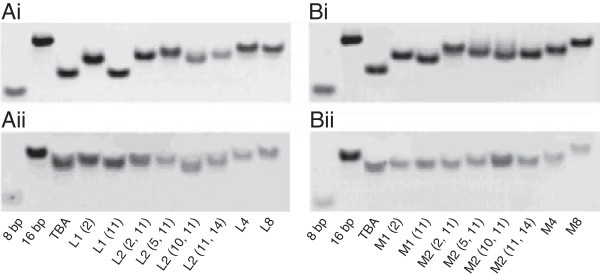
**PAGE images of the native TBA and TBAs substituted with LNA (left) or OMeNA (right) in 50 mM KCl (upper) or 50 mM CaCl**_**2 **_**(lower) electrophoresis buffers, respectively.** A, LNA-modified TBAs in 50 mM KCl (Ai) and 50 mM CaCl_2_ (Aii); B, OMeNA-modified TBAs in 50 mM KCl (Bi) and 50 mM CaCl_2_ (Bii). Native polyacrylamide gel electrophoresis was run in 50 mM KCl (upper) or 50 mM CaCl_2_ (lower) electrophoresis buffers, respectively. Lanes 1 and 2, markers for 8 bp (C_4_G_4_*C_4_G_4_), and 16 bp (G_3_TG_4_AG_3_TG_3_*C_3_AC_3_TC_4_AC_3_) base-pair DNA duplexes, respectively; lane 3 and others, TBA and modified-TBAs loaded at 20 μM nucleoside concentrations, respectively.

Although it has been reported that monovalent K^+^ ions can stabilize the TBA architecture effectively [13], the effects of divalent ions have been much less well explored. Here, we compared the TBA structure in different ionic environments (some data not shown). Figure 2D, E and F show the CD spectra of native TBA and modified TBAs in 50 mM Ca^2+^ buffer. In Ca^2+^ buffer, the most striking result is that the native TBA and two L1 TBAs were almost unstructured (Figure 2D). Interestingly, L2 TBAs, except L2 (2, 11), presented a broad positive CD absorbance at 265 nm and a minimum at 240 nm (Figure 2E), i.e. the spectral signature of a parallel G-quadruplex [6,18]. Furthermore, four and eight LNA-substituted TBAs also exhibited characteristic CD profiles of parallel G-quadruplexes (Figure 2F), while OMeNA, M1, M2 and M4 TBAs were still unstructured as native TBA in general (Figure 2D and E), and only M8 TBA was induced to form a perfect parallel G-quadruplex structure (Figure 2F). These data clearly demonstrate that, in 50 mM Ca^2+^ buffer, the native TBA lost its native anti-parallel G-quadruplex structure; however, LNA- or OMeNA substitutions could transform it into a parallel G-quadruplex conformation. In the meantime, we analyzed all of these TBAs using non-denaturing PAGE electrophoresis to confirm the molecular identity of the conformation. In comparison with 8 bp and 16 bp oligomers as mobility markers, the native TBA in the K^+^ environment migrated at a rate that was equivalent to a 10 bp oligomer. The substituted TBAs moved differently due to their topological variations, and the unstructured TBA moved at a rate that was similar to a 14 bp oligomer (Figure 3Ai and Bi). In the Ca^2+^ environment, substitutions further retarded TBA migration, particularly those of L8 and M8 TBAs (Figure 3Aii and Bii). This indicated the co-existence of different homodimeric forms, leading us to speculate that the parallel TBA G-quadruplex in Ca^2+^ environment might be in a dimeric form, and propose two folding topologies of the bimolecular parallel TBA G-quadruplex (Additional file 1: Figure S2).

## Discussion

Oligonucleotide modifications have attracted great attention in structural and functional research, in which LNA and OMeNA are quite widely used in aptamer modifications. Previous studies have shown that single LNA-substitutions for G2, T4, G5, T7, G8 or G15 have no effect on TBA structural alterations [19,20], and that LNA-substituted TBAs on two G-tetrad planes could have different structures coexisting in solution [20]. These results might be correlated with glycosidic bond angles determined by the positions where LNA nucleotides were incorporated, as it is the preference of LNA nucleotides to adopt an anti-glycosidic conformation based on the C3′-endo sugar pucker [21]. OMeNA-substituted TBAs, however, have been rarely studied before. In this study, we report for the first time that structural transitions of TBA are regulated by multisite-specific LNA or 2′-OMeNA substitutions. The results showed that the native structure of TBA changed from an anti-parallel G-quadruplex to an unstructured form in a K^+^ environment, and from an unstructured form to a parallel G-quadruplex in a Ca^2+^ environment. The molecular mechanism may involve the syn- or anti-conformation of guanine residues of G-quartet construction (Additional file 2: Figure S1), and optional modified sites (except for ion effect). Also, Additional file 1: Figure S2 shows the assumed possible conformations of multi-LNA- or OMeNA-modified TBAs. These results suggest that the G-quadruplex conformation mostly depends on the glycosidic conformation of guanine residues in the G-tracts, which corresponds with a previous report [21]. Otherwise, different metal ions may also play an unneglected role in G-quadruplex structure formation [22]. Accordingly, specific regulatory mechanisms of G-quadruplex conformation may exist, by which the current experimental evidence provides us an opportunity to create polymorphic G-quadruplexes using nucleotide derivative substitutions at selected positions in conjunction with different ionic conditions.

## Conclusions

In summary, we monitored diverse quadruplex structural transformations through LNA or OMeNA site-specific modifications on TBA, which were regulated by the syn- or anti-conformation of guanine residues of G-quartet construction and optional modified sites. Moreover, both LNA and OMeNA substitutions could alter the TBA G-quadruplex structure, and OMeNA might have more destructive power than LNA to convert TBA into a structureless form in a K^+^ environment; in a Ca^2+^ environment, LNA- or OMeNA substitutions could transform the native unstructured TBA into a parallel G-quadruplex conformation. These observations enrich our understanding of the nature of LNA and OMeNA substitutions and the behavior of G-quadruplex structure formation. The significance of these structural transitions may benefit the regulation of gene expression (i.e. manual intervention of gene therapy), genetic analyses, molecular diagnosis and the construction of nano-scale biostructures.

### Experimental

#### Preparation of oligonucleotides

The oligonucleotides were purchased from TaKaRa Biotech Ltd. (Dalian China). The lyophilized oligonucleotides were dissolved in 1 × TE buffer (10 mM Tris–HCl and 1 mM EDTA, pH 7.2) to give a stock solution concentration of 100 μM. Before starting the experiments, all of the oligonucleotide samples were denatured (5 min at 95°C) to remove aggregates. The sample was then left to cool to room temperature. The formation of quadruplexes was followed at 4°C, unless stated otherwise.

#### Circular dichroism spectroscopy

Circular dichroism measurements were carried out on a Jasco J-810 spectropolarimeter (Jasco, Easton, MD) in a quartz sample cell with an optical path length of 1 mm. 500 μL of sample solution was added and the cell was placed in a thermostable holder maintained at room temperature during the measurements. The CD spectra were representative of three averaged scans at a speed of 500 nm per minute, made from 340 nm to 220 nm. The final DNA concentration of 5 μM for the CD spectroscopic study was prepared in 1 × TE buffer (10 mM Tris–HCl and 1 mM EDTA, pH 7.2) with either 50 mM KCl or CaCl_2_, respectively. To ensure that all TBA aptamers adopted the same structure at the beginning of each experiment, a slow melting-cooling cycle at 0.2°C per minute was performed prior to each experiment. The unsubstituted TBA and modified-TBAs with identical sequences were assumed to have identical extinction coefficients.

#### Non-denatured PAGE electrophoresis

Different substituted TBA species were incubated with 1 × TE buffer (10 mM Tris–HCl and 1 mM EDTA, pH 7.2) in the presence of 50 mM KCl or CaCl_2_ at 37°C for 1 h, respectively. The 15% non-denatured PAGE gels were prepared by mixing 10 mL 30% acrylamide solution, 4 mL 5 × TB buffer (containing 250 mM KCl or CaCl_2_), 5.86 mL water, 140 μL 10% APS, and 13 μL TEMED. The 8 × 10 × 0.1 cm gel was polymerized within 45 min. A final concentration of 20 μM TBA solution (containing 2 μL stock solution, 2 μL loading buffer, 0.5 μL 1 M KCl or CaCl_2_, and 5.5 μL water) was loaded onto gels. Gels were run in 1 × TB buffer (containing 50 mM KCl or 50 mM CaCl_2_) for 1.5 h at 100 V at 4°C. The gels were then stained with Stains-All (Sigma, USA), washed three times, and images were recorded using a Personal Scanner (Model Z320, FangZheng, China).

## Abbreviations

LNA: Locked nucleic acid; OMeNA: 2*'*–O-methyl nucleic acid; TBA: Thrombin binding aptamer; CD: Circular dichroism; TE: Tris–HCl EDTA; TB: Tris-boronic acid; EDTA: Ethylene Diamine Tetraacetic Acid; APS: Ammonium persulfate; TEMED: Tetramethylethylenediamine; PAGE: Polyacrylamide gel electrophoresis.

## Competing interests

The authors declare that they have no competing interests.

## Authors’ contributions

BL and DL conceived of the study, participated in its design and drafted the manuscript. BL and DL carried out data acquisition, interpretation and performed the statistical analysis. All authors read and approved the final manuscript.

## Supplementary Material

Additional file 1: Figure S2 The proposed folding topologies of all TBAs used in this study in 50 mM K^+^ (A) or Ca^2+^ (B). Click here for file

Additional file 2: Figure S1 The schematic diagram of the syn- or anti-glycosidic conformation of modified guanine residues in a G-quartet of a parallel (A) or anti-parallel (B) G-quadruplex. Click here for file
