# Supplementary figures and images for: Structural transformation induced by locked nucleic acid or 2′–O-methyl nucleic acid site-specific modifications on thrombin binding aptamer
Source: Chem Cent J. 2014 Mar 19;8:19. doi: 10.1186/1752-153X-8-19 (PMC4000052; doi:10.1186/1752-153X-8-19)

Supplementary Figure 2

A

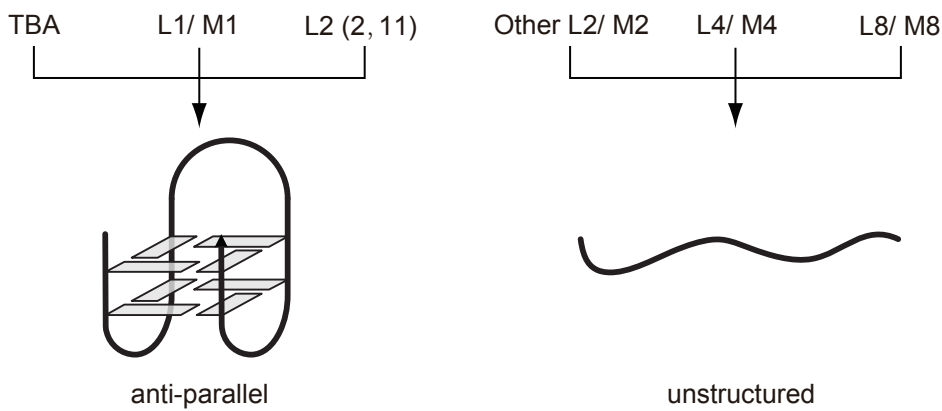

B

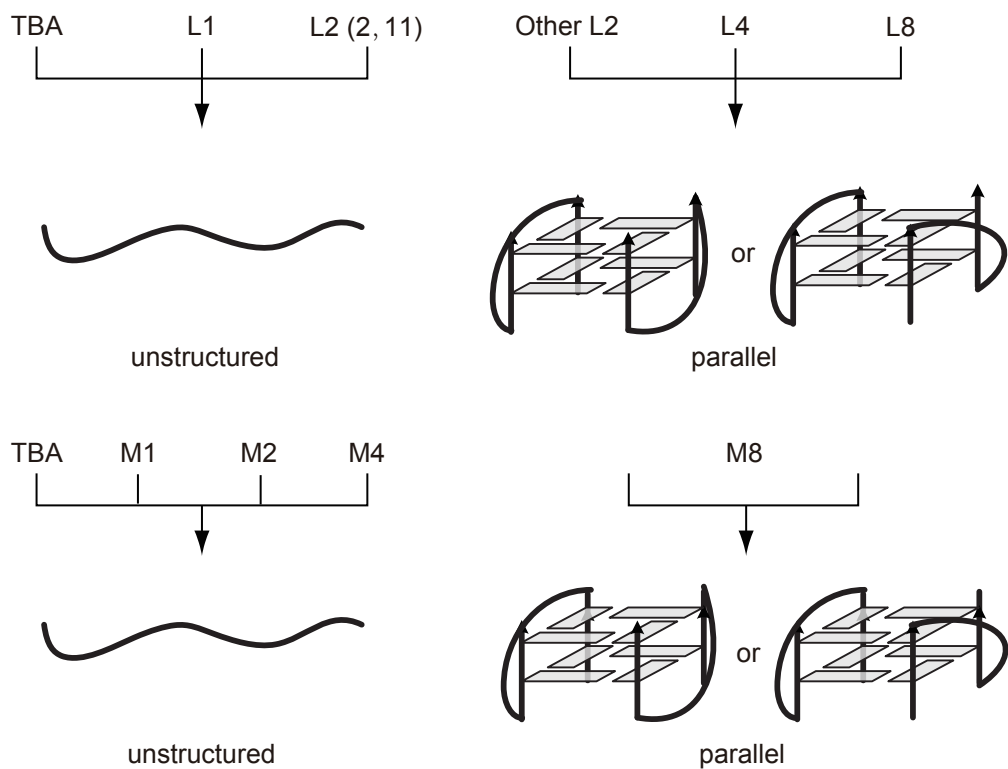

Supplement: Additional file 1: Figure S2 — The proposed folding topologies of all TBAs used in this study in 50 mM K+ (A) or Ca2+ (B). [file 1752-153X-8-19-S1.pdf]

Supplementary Figure 1

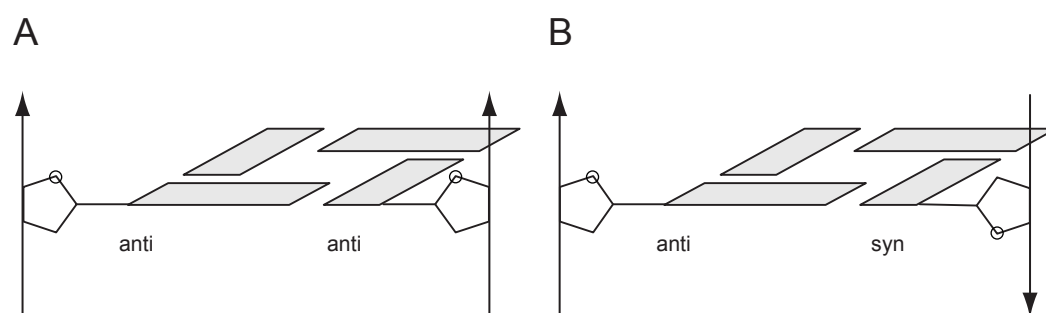

Supplement: Additional file 2: Figure S1 — The schematic diagram of the syn- or anti-glycosidic conformation of modified guanine residues in a G-quartet of a parallel (A) or anti-parallel (B) G-quadruplex. [file 1752-153X-8-19-S2.pdf]
